# A comparative study of EEG microstate dynamics during happy and sad music videos

**DOI:** 10.3389/fnhum.2024.1469468

**Published:** 2025-02-06

**Authors:** Ashish Gupta, Chandan Kumar Srivastava, Braj Bhushan, Laxmidhar Behera

**Affiliations:** ^1^Department of Electrical Engineering, Indian Institute of Technology, Kanpur, India; ^2^Department of Humanities and Social Sciences, Indian Institute of Technology, Bombay, India; ^3^Department of Humanities and Social Sciences, Indian Institute of Technology, Kanpur, India; ^4^School of Computing and Electrical Engineering, Indian Institute of Technology, Mandi, India

**Keywords:** EEG microstate, emotion, music, attention, mind wandering

## Abstract

EEG microstates offer a unique window into the dynamics of emotional experiences. This study delved into the emotional responses of happiness and sadness triggered by music videos, employing microstate analysis and eLoreta source-level investigation in the alpha band. The results of the microstate analysis showed that regardless of gender, participants during happy music video significantly upregulated class D microstate and downregulated class C microstate, leading to a significantly enhanced global explained variance (GEV), coverage, occurrence, duration, and global field power (GFP) for class D. Conversely, sad music video had the opposite effect. The eLoreta study revealed that during the happy state, there was enhanced CSD in the central parietal regions across both genders and diminished functional connectivity in the precuneus for female participants compared to the sad state. Class D and class C microstates are linked to attention and mind-wandering, respectively. The findings suggest that (1) increased class D and CSD activity could explain heightened attentiveness observed during happy music, and (2) increased class C activity and functional connectivity could explain enhanced mind wandering observed during sad music. Additionally, female participants exhibited significantly higher mean occurrence than males, and the sad state showed significantly higher mean occurrence than the happy state.

## 1 Introduction

MUSIC is evolutionary linked to human brains (Cross and Morley, 2008) in as much as humans can readily recognize basic emotions such as happiness and sadness (Brattico et al., 2011). Apart from improving one's mood, music has been utilized to achieve various self-regulatory objectives. Listening to happy music is linked to improved cognitive functions such as attention (Gupta et al., 2018; Putkinen et al., 2017) and spatial-temporal abilities, sometimes referred to as the “Mozart effect” (Gupta et al., 2018; Putkinen et al., 2017; Rauscher et al., 1995; Wilson and Brown, 1997). Conversely, sad music aids in emotional processing and introspection, offering comfort and fostering emotional resilience during challenging times (Van den Tol et al., 2016; Van den Tol and Edwards, 2013).

However, music research faces challenges, including the lack of a scientifically standardized approach to music administration, the reduction of music's effects to superficial aesthetic or mood-related features, and limited understanding of the brain's dynamic during music listening. Addressing these challenges requires precise analyses to fully explore music's impact on cognitive domains such as attention and intelligence. This research has the potential to transform approaches to mental health, education, and cognitive rehabilitation, promoting wellbeing through accessible, non-invasive methods.

Attention is a fundamental cognitive function that enables us to selectively focus on specific stimuli, tasks, or thoughts while filtering out irrelevant information (Callan et al., 2023). Research indicates that attention is shaped by prior context (Mugruza-Vassallo et al., 2021) and the emotional significance of stimuli or events (Bröckelmann et al., 2011). The early auditory processing, in turn, is modulated by attention (Karns and Knight, 2009). Studies show that even brief exposure to happy music can activate brain regions linked to memory, attention, and IQ, while also minimizing unnecessary brain activity, leading to optimized cognitive efficiency. Similarly, sad music aids in the achievement of various self-regulation goals in the domains of cognition, social, memory retrieval, friend, distraction, mood enhancement, and re-experience affect (Van den Tol and Edwards, 2013; Van den Tol et al., 2016) ultimately leading to better emotional and memory processing, especially during difficult situations (Gupta et al., 2023).

Taruffi et al. (2017), who specifically explored the impact of happy and sad music on mind wandering and meta-awareness, found that happy music significantly enhanced meta-awareness compared to sad music, whereas sad music led to a significant increase in mind wandering compared to happy music. However, the mind-wandering experience while listening to sad music is distinct from that of ordinary sadness and is uniquely characterized by the melancholic yet pleasurable nature of sad music (Gupta et al.,^2023^; Taruffi and Koelsch, 2014; Sachs et al., 2015).

The brain associations of basic emotions of happiness and sadness in music have been explored in only a limited number of studies. One of the initial studies (Khalfa et al., 2005), using functional magnetic resonance imaging (fMRI), found that sad music stimulated the left medial frontal gyrus and the adjacent superior frontal gyrus, more as compared to happy music. These brain regions are linked to emotional experiences, self-reflection, and self-evaluation (Jacobsen et al., 2006; Kornysheva et al., 2010). fMRI maps brain activity by detecting blood flow changes tied to neural activity. It provides high spatial resolution, helping identify brain regions involved in cognition and emotion, although its temporal resolution is limited (Varvatsoulias, 2013).

fMRI studies have also shown that compared to neutral composition, happy music activates several brain regions such as the superior frontal gyrus, anterior cingulate cortex, posterior cingulate gyrus, parahippocampal gyrus, medial frontal gyrus, and precuneus (Mitterschiffthaler et al., 2007), while sad music activates brain regions such as the hippocampus/amygdala, posterior cingulate gyrus, medial frontal gyrus, and cerebellum (Mitterschiffthaler et al., 2007). However, there is need to investigate brain activity particularly during basic primary emotion of happiness and sadness evoked by music using EEG especially in connection to cognitive and emotion processing.

Understanding how the brain processes information has led to extensive research on large-scale resting-state brain networks, focusing on their spatial structure and temporal dynamics. A key method in this research is the analysis of EEG microstates, which represent snapshots of the brain's global neuronal activity. It represent episodes of synchronized electrical activity in the brain that last for tens of milliseconds (Michel and Koenig, 2018) and illustrate how specific spatial and temporal configurations of neuronal activity align with mental processes or the resting state of the brain (Michel and Koenig, 2018; Lehmann and Michel, 2011).

Further investigations have found consistent and specific spatio-temporal brain microstates across independent studies (Khanna et al., 2015; Michel and Koenig, 2018), making them potential markers of neural traits (Schiller et al., 2020). These functional microstates are usually identified as four prototypical microstates termed class A, class B, class C, and class D and are known for auditory processing, visual processing, default mode network (DMN), and attention respectively (Khanna et al., 2015; Michel and Koenig, 2018; Koenig et al., 2002). Studies have shown that disruptions in cognitive processes related to psychiatric and neurological disorders are linked to changes in the temporal dynamics of these microstates (Soni et al., 2019; Michel and Koenig, 2018).

Microstate analysis has been used in a wide range of studies, including resting state of the brain (Schiller et al., 2020), neuropsychiatric diseases (Nishida et al., 2013), sleepiness (Cantero et al., 1999), gender differences (Tomescu et al., 2018), and tasks-based brain activities (Seitzman et al., 2017; Hu et al., 2023).

Unlike emotional states, which change gradually over time, EEG signals are unsteady and change rapidly, resulting in highly variable extracted features. As a result, Chen et al. (2021) argue that analyzing EEG microstates can offer deeper insight into emotional research than traditional EEG analysis and better capture the spatial-temporal characteristics of spontaneous brain activity under varying emotional states. Indeed, microstate analysis has been used successfully in emotional research (Prete et al., 2022; Chen et al., 2021; Coll et al., 2019) and has the potential to improve emotion classification (Chen et al., 2021; Shen et al., 2020). Studies reveal that the four EEG microstates are proficient in capturing the dynamic features of emotions (Prete et al., 2022; Hu et al., 2023).

A recent review has shown it as an effective tool for investigating socio-affective states (Schiller et al., 2024) and emotional processing (Schiller et al., 2024), providing a dynamic whole-brain representation of distinct emotions (Liu et al., 2023). Specifically, research investigating the impact of music on the brain microstate shows improved microstates related to speech, vision, and attention processing (Jiang and Zheng, 2024) in participants who are trained in music as compared to untrained participants. Microstate analysis has also advanced our understanding of the neural mechanisms underlying the effectiveness of music therapy for tinnitus (Zhu and Gong, 2023). Furthermore, happy music can modify brain microstates, leading to positive effects on cognitive reappraisal (Hua and Li, 2023).

In this current investigation, we used the widely recognized DEAP database (Koelstra et al., 2011), specifically designed for emotion analysis using physiological signals. A recent microstate analysis of the DEAP dataset highlighted the effectiveness of alpha band microstates in accounting for variances across all EEG time frames, surpassing other frequency bands (Shen et al., 2020). Remarkably, the microstate topologies within the alpha band closely resembled the four maps previously identified more than those in other bands (Shen et al., 2020). Additionally, the concept of microstates was first applied to alpha oscillations in the 1987 (Lehmann et al., 1987), and recent studies have confirmed that alpha-band activity is the prominent driver of microstates (Milz et al., 2017). Several other studies have also shown the alpha band to play vital roles in cognitive functions during music listening (Wu et al., 2012; Flores-Gutiérrez et al., 2009; Gupta et al., 2018, 2023). Therefore, in our current analysis of the DEAP dataset, we focused our investigation on the alpha band. This is also in line with the earlier microstate studies (Gu et al., 2022; Das et al., 2024).

A prior investigation using the DEAP database identified four as the optimal cluster number (Hu et al., 2023). In our current analysis, we used the same DEAP dataset. Consequently, we selected four microstates for our study. Four microstates are the most consistent observed and studied across different research studies and provide clear neurophysiological interpretations linked to various human cognitive functions. This is in line with earlier studies (Al Zoubi et al., 2019; da Cruz et al., 2020; Koenig et al., 2002).

In prior EEG, studies delving into neural signatures for emotions, particularly in the alpha band, have demonstrated an increase in EEG power, in the central-parietal regions during passive listening to music (Markovic et al., 2017; Jäncke et al., 2015). This phenomenon is linked to heightened attentiveness (Markovic et al., 2017; Jäncke et al., 2015) with the results also indicating a positive correlation with valence (Koelstra et al., 2011). Studies focusing on internal tasks such as self-referential process (Knyazev, 2013), meditation (Aftanas and Golocheikine, 2001), and music listening (Markovic et al., 2017; Jäncke et al., 2015) had shown that alpha band oscillations (power) to be directly proportional to cortical activity within the task relevant area.The examination of functional connectivity in brain networks revealed heightened connectivity, particularly in the alpha band, during music listening (Wu et al., 2012; Flores-Gutiérrez et al., 2009; Gupta et al., 2023).

Gender is an important factor to consider while studying the brain's response to basic emotions (Stevens and Hamann, 2012), and in general, females had a greater brain activity than males (Goshvarpour and Goshvarpour, 2019). In this study, we also aim to investigate the role of gender differences in processing musical stimuli while also accounting for valence as contributing factor.

Thus, the current study investigates the brain microstates underlying basic emotions of happiness and sadness in the alpha band for male and female participants. As discussed earlier, Taruffi et al. (2017) found that relatively happy music significantly boosts meta-awareness more than sad music, while sad music increases mind wandering more than happy music. These findings remained consistent across multiple experiments investigating the effects of happy (Gupta et al., 2018; Putkinen et al., 2017) and sad music (Gupta et al., 2023; Taruffi and Koelsch, 2014; Sachs et al., 2015). The DMN has been identified as the primary network involved in mind-wandering (Mason et al., 2007; Kucyi et al., 2013). Consequently, we hypothesize that sad music would influence the class C microstate, associated with DMN activity, while happy music would affect the class D microstate, linked to attention. We performed source reconstruction analysis through eLoreata to further investigate the brain regions underpinning emotional experience and expect enhanced brain activity during listening to happy music compared to sad one.

## 2 Methods

### 2.1 Procedure and EEG data

The study utilized the DEAP dataset (Koelstra et al., 2011), which is an open-source EEG dataset consisting of recordings from 32 participants (17 male) with mean age of 27.18 (SD = 4.44) listening to 40 musical videos, each lasting 1 min. Data were recorded at two locations: Participants 1–22 in Twente and 23–32 in Geneva. The DEAP database utilized music-video clips to evoke emotional responses in subjects. Before commencing the emotional experiment, a 2-min baseline recording was taken. During this time, subjects were instructed to relax, while a fixation cross was displayed. Subsequently, 40 videos were presented across 40 trials. The musical clip presentations were randomized for each participant. Each trial began with the display of the trial number for 2 s, indicating the subject's progress, followed by a 5-s fixation cross. Then, the music video was shown for 1 min, after which the subject completed a self-assessment. A brief break was provided after the 20th trial, during which volunteers were offered non-caffeinated and non-alcoholic beverages and cookies, and the examiner checked the signal quality and electrode placement. The second half of the experiment was then conducted. Participants rated their experience on valence, arousal, dominance, liking, and familiarity scales from 1 to 9. EEG was recorded from 32 channels based on the standard 10–20 system of electrode placement, with a sampling frequency of 512 Hz. Further details can be found in Koelstra et al. (2011).

The musical stimuli were selected based on ratings for arousal, valence, dominance, and the Genova emotion scale (Koelstra et al., 2011). In the current study, we only selected stimuli which had significantly expressed the respective emotions of happiness and sadness, based upon the Genova scale rating. We identified one music video, with the ID number 11, that received significant ratings for happiness, and another one, with the ID number 30, that was significantly rated for sadness (see Supplementary Figures S1, S2). Therefore, these specific videos were chosen for the current investigation. Other music videos did not exhibit significant expressions of happiness or sadness.

Koelstra et al.'s work concentrated on traditional EEG power analysis of scalp potentials, primarily examining valence and arousal within a dimensional framework. The present study expands this investigation by incorporating (1) global neural activity assessment through microstate analysis, (2) source-level analysis using eLoreta, and (3) evaluation of music videos based on discrete emotion theory.

### 2.2 EEG pre-processing

The EEG data were down-sampled to 256 Hz and visually checked for artifacts. Bad electrodes were marked and interpolated. The EEG data were re-referenced to average reference in line with earlier studies (Gupta et al., 2023; Goshvarpour and Goshvarpour, 2019; Koelstra et al., 2011; Hu et al., 2023). To further remove eye and muscle movement artifacts, independent component analysis (ICA) and SASICA were employed after rank adjustment. The EEGLAB toolbox was utilized for implementing ICA and SASICA, which have proven effectiveness in eliminating artifacts associated with eyes and muscle movements (Sburlea et al., 2021; Khosravani et al., 2019). EEG data were filtered between 8 and 13 Hz to obtain the alpha band. We analyzed the EEG data under four conditions: (1) Female during listening to happy music (FH), (2) Female during listening to sad music (FS), (3) Male during listening to happy music (MH), and (4) Male during the listening of sad music (MS).

### 2.3 Microstate analysis

A spatial k-means cluster analysis, as implemented in the EEGLAB toolbox (Poulsen et al., 2018), was applied separately for FH, FS, MH, and MS conditions. The cluster analysis was performed using maps at the local maxima of the global field power (GFP), which represents the time points with the highest signal to noise. The polarity of the maps was not considered. Microstate cluster analysis was performed on the concatenated EEG data of the participants under each condition. We extracted four microstates for each condition (Bréchet et al., 2020; da Cruz et al., 2020; Tait et al., 2020). Koenig et al. (1999) categorized four microstate maps of the brain into classes A, B, C, and D based upon the topological orientation of the map. Specifically, microstate map A displays a left-right orientation, map B exhibits a right-left orientation, map C demonstrates an anterior-posterior orientation, and map D reveals a fronto-central maximum. Subsequent studies have consistently maintained this labeling convention (Michel and Koenig, 2018) (Supplementary Figures S3–S5). We categorized the acquired microstates in our study as classes A, B, C, and D based on their topographical orientation, as outlined by Koenig et al. (1999), in line with earlier studies (Hu et al., 2023; Pal et al., 2021; Liu et al., 2021; Pascual-Marqui et al., 2014). Furthermore, we calculated the spatial correlation among the four microstates of the brain under the four conditions. After identifying the maps for each condition, the maps were fitted back to the EEG data of each participant under each condition. Each time frame was assigned to templates that best fit the data in terms of spatial correlation. This process resulted in a microstate sequence for each participant, which was then used to calculate the microstate parameters specific to each participant for each condition.

(1) GEV: It is a parameter that measures how well the chosen template maps describe the entire dataset.
(2) Coverage: Coverage of microstates indicates the percentage of the specified microstates in the total recorded time.
(3) Occurrence: Frequency of occurrence measures the average number of times the microstate occurs per second.
(4) GFP: Global field power is a measure of the strength of the electric field generated by the brain at any instant of time.
(5) Transition probability: Transition probability between different microstates is the likelihood of transitioning from the current microstate to another state.
(6) Duration: It refers to the average length of time a specific microstate remains dominant.

### 2.4 EEG source analysis

eLoreta is a source localization method that uses a weighted minimal norm inverse technique to perform three-dimensional source localization (Pascual-Marqui et al., 2011). It offers exact localization (zero localization error) using a discrete, distributed, and linear approach and was used to analyze the EEG data in this study. EEG current source density (CSD) refers to the estimation of the electrical current flow within the cortex, based on scalp EEG recordings. CSD provides a measure of the intensity and distribution of active neural sources by calculating the spatial second derivative of the EEG potential. CSD was computed at 6,239 voxels, with a sampling resolution of 5 mm, using eLoreta software.The study used functional connectivity as a tool to investigate how brain regions synchronize to accomplish tasks. Lag phase synchronization was selected as the metric of interest as it measures non-linear functional connectivity while accounting for factors such as power fluctuations, instantaneous zero lagged components, and volume conduction. This choice was made to ensure resistance to non-physiological artifacts and enhance the validity of the findings (Pascual-Marqui et al., 2011). Brain connectivity between all pairs of the standard 68 regions of interest (ROI) defined by the Desikan-Killiany atlas was computed at the source level using eLoreta (Supplementary Table S1).

### 2.5 Statistical analysis

To analyze the data, we utilized a two-tailed *t*-test with a significance level (α) of 0.05 for comparing mean values and subjective questionnaires. To examine the influence of gender, microstates, and stimulus type on parameters such as GEV, occurrence, GFP, duration, and coverage, a three-way analysis of variance (ANOVA) was conducted using SPSS software. In this analysis, gender was treated as a between-subjects factor, meaning it varied across different participants, allowing us to assess if there are differences in these parameters between male and female participants. Meanwhile, microstates and stimulus type were included as within-subjects factors as each participant experienced different microstates and stimulus conditions. This three-way ANOVA enabled us to determine not only the main effects of each factor (gender, microstates, and stimulus type) on the parameters but also any interaction effects between them, showing how combinations of these factors may influence the outcomes in complex ways. To account for multiple testing across microstates and stimuli, we applied false discovery rate (FDR) correction.

eLoreta source-level data analysis at the each voxel presents issues with multiple testing. To address this, eLoreta uses the non-parametric SnPM method, performing 5,000 randomizations to establish accurate probability thresholds, correcting for multiple comparisons without relying on normal distribution assumptions. SnPM is implemented in the eLoreta statistical package (Holmes et al., 1996). SnPM has been widely validated, enhancing reliability in EEG source analysis (Pascual-Marqui et al., 1999).

## 3 Results

### 3.1 Specific microstate maps for each state

We obtained four microstates maps explaining together 69.97, 69.46, 68.27, and 70.30 percent GEV for MH, MS, FH, and FS states, respectively.

**Figure 1:**
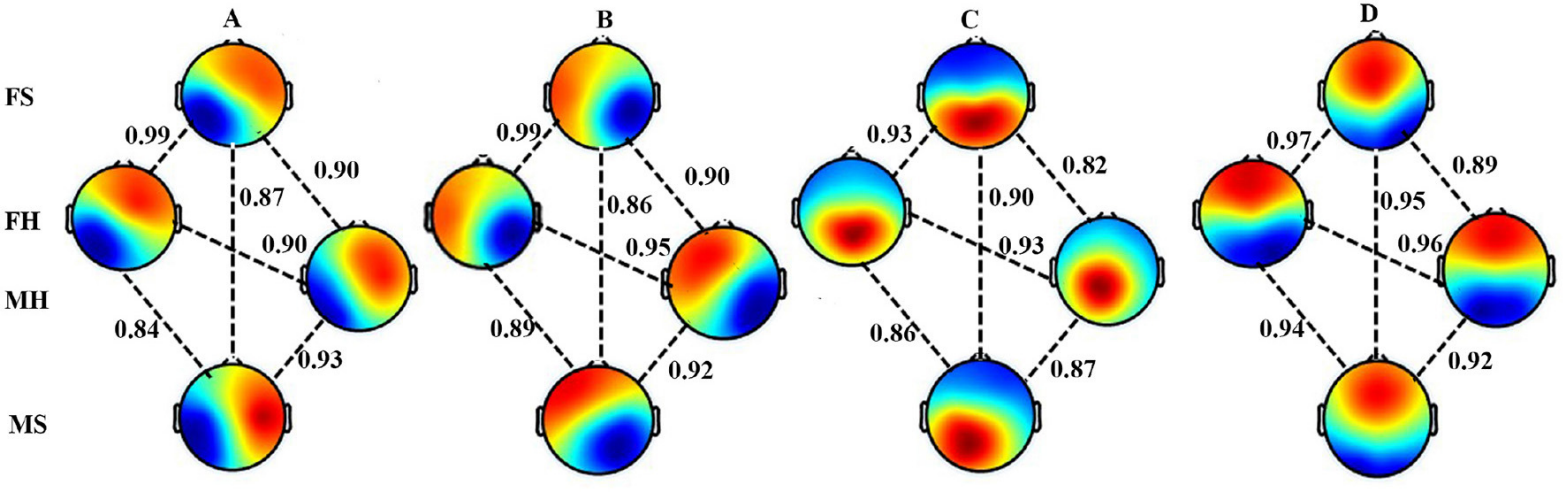
Microstate maps. Four EEG microstates under FS, FH, MS, and MH conditions. Spatial correlation between the corresponding microstate class across conditions.

Figure 1 shows the four microstate maps under each condition categorized as per standard convention into the four Class A, B, C, and D based upon the highest spatial correlation and visual inspection (Hu et al., 2023; Pascual-Marqui et al., 2014). Figure 1 shows high spatial correlations among the different conditions for the corresponding microstate category A-D (*p* < 0.0001). This confirms that the respective microstates across the four conditions for each class are consistently aligned among themselves, representing the same microstate type.

### 3.2 Microstate parameters

The microstate maps were fitted back into the EEG data of the participants under each condition to obtain several parameters such as GEV, coverage, occurrence, duration, and inter-microstate transition probability.

**Figure 2:**
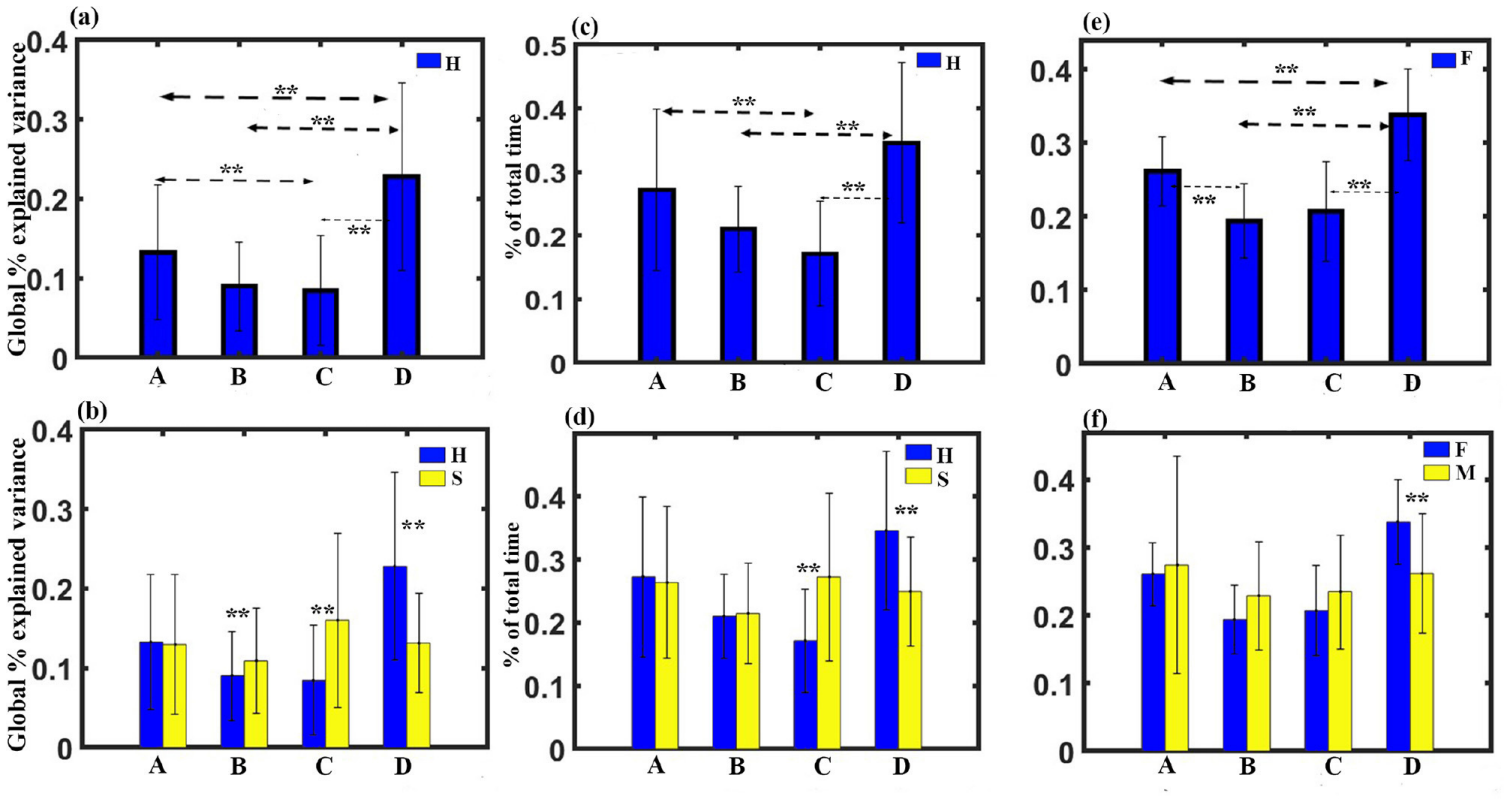
Microstate parameters. **(A)** Relative GEV of microstates during Happy stimulus across gender. **(B)** Relative GEV in each microstate during Happy and Sad stimulus across gender. **(C)** Relative coverage of microstates during Happy stimulus across gender. **(D)** Relative coverage in each microstate during Happy and Sad stimulus across gender. **(E)** Relative coverage of microstates during female participants across stimulus. **(F)** Relative coverage of microstates for female and male participants across stimulus (**FDR corrected, *p* < 0.05, error bars = 1 SD).

(1) GEV analysis: We administered a three-way ANOVA with gender as in between factor, stimulus, and microstate as within factor. The results show no significant three-way interaction. We obtained a significant two-way interaction between stimulus and microstate with a Greenhouse-Geisser correction (*F*_1.522, 45.662_ = 13.438, *p* < 0.001). To examine the simple effect of microstates, a one-way repeated measures ANOVA was conducted. Findings revealed a significant effect of microstates on GEV for happy stimulus with a Greenhouse-Geisser correction (*F*_2.177, 67.492_ = 15.440, *p* < 0.001). We did not obtain any significant effect of microstate for sad stimulus through one-way repeated measure ANOVA. Further *post-hoc* pairwise comparison with FDR correction revealed that regardless of gender, class D state to be significantly higher than class C (t = 5.0036, df = 31, *p* < 0.0001, effect size = 0.9201), class B (t = 5.6165, df = 31, *p* < 0.0001, effect size = 0.7294), and class A (t = 3.1354, df= 31, *p* < 0.005, effect size = 0.3290) during the happy stimulus. We also found class C state to be significantly reduced GEV than class A (t = −2.2977, df = 31, *p* < 0.05, effect size = −0.8899) as shown in Figure 2A. We obtained microstate class C and class D during the sad stimulus to be significantly higher and lower than the class C (t = 3.1266, df = 31, *p* < 0.005, effect size = 0.5527) and class D (t = −4.9658, df = 31, *p* < 0.001, effect size = −0.8778), respectively, during the happy stimulus as shown in Figure 2B. We also observed microstate class B during the sad stimulus to be significantly higher than the class B (t = 2.3415, df = 31, *p* < 0.05, effect size = 0.4139) during the happy stimulus.

(2) Coverage analysis: A three-way ANOVA with gender as in between factor, stimulus, and microstate as within factor was administered. The results show no significant three-way interaction. We obtained a significant two-way interaction between stimulus and microstate with a Greenhouse-Geisser correction (*F*_1.441, 43.225_ = 12.609, *p* = 0.001). Further one-way repeated measure ANOVA was conducted to examine the simple effect of microstates. Findings revealed a significant effect of microstates on coverage for happy stimulus with a Greenhouse-Geisser correction (*F*_2.125, 65.886_ = 12.974, *p* < 0.001).

We also obtained a significant two-way interaction between gender and microstate with a Greenhouse-Geisser correction (*F*_1.441, 43.225_ = 12.609, *p* = 0.001). Further one-way repeated measure ANOVA was done to investigate the simple effect of microstates. Findings revealed a significant effect of microstates on coverage for happy stimulus for female participants with a Hyunh-Feldt correction(*F*_2.751, 38.508_ = 14.767, *p* < 001).

*Post-hoc* pairwise comparison with FDR correction revealed that regardless of gender, class D state to be significantly higher than class C (t = 5.3447, df = 31, *p* < 0.001, effect size = 0.9448) and class B (t = 5.2380, df = 31, *p* < 0.001, effect size = 0.9260) during the happy stimulus. We also found class C state to be significantly reduced coverage than class A (t = −3.5234, df= 31, *p* < 0.005, effect size = −0.6229) as shown in Figure 2C. We obtained microstate class C and class D during the sad stimulus to be significantly higher and lower than the class C (t = 3.6066, df = 31, *p* < 0.005, effect size = 0.6376) and the class D (t = −4.0892, df= 31, *p* ≤ 0.001, effect size = −0.7229), respectively, during the happy stimulus as shown in Figure 2D.

Pairwise comparison further showed that regardless of stimulus, class D was significantly higher than class A (t = 3.6063, df = 14, *p* ≤ 0.001, effect size = 0.9311), class B (t = 5.2938, df = 14, *p* ≤ 0.001, effect size = 1.3669), and class C (t = 4.9116, df = 14, *p* ≤ 0.001, effect size = 1.2682) for female participants as shown in Figure 2D. We obtained class B to be significantly reduced that class A (t = −4.5118, df = 31, *p* ≤ 0.001, effect size = −1.1650). Two sample t-test further showed that Class D during female participants was significantly enhanced compared to class D (t = −4.0892, df = 31, *p* ≤ 0.001, effect size = −0.7229) during male participants as shown in Figure 2E.

**Figure 3:**
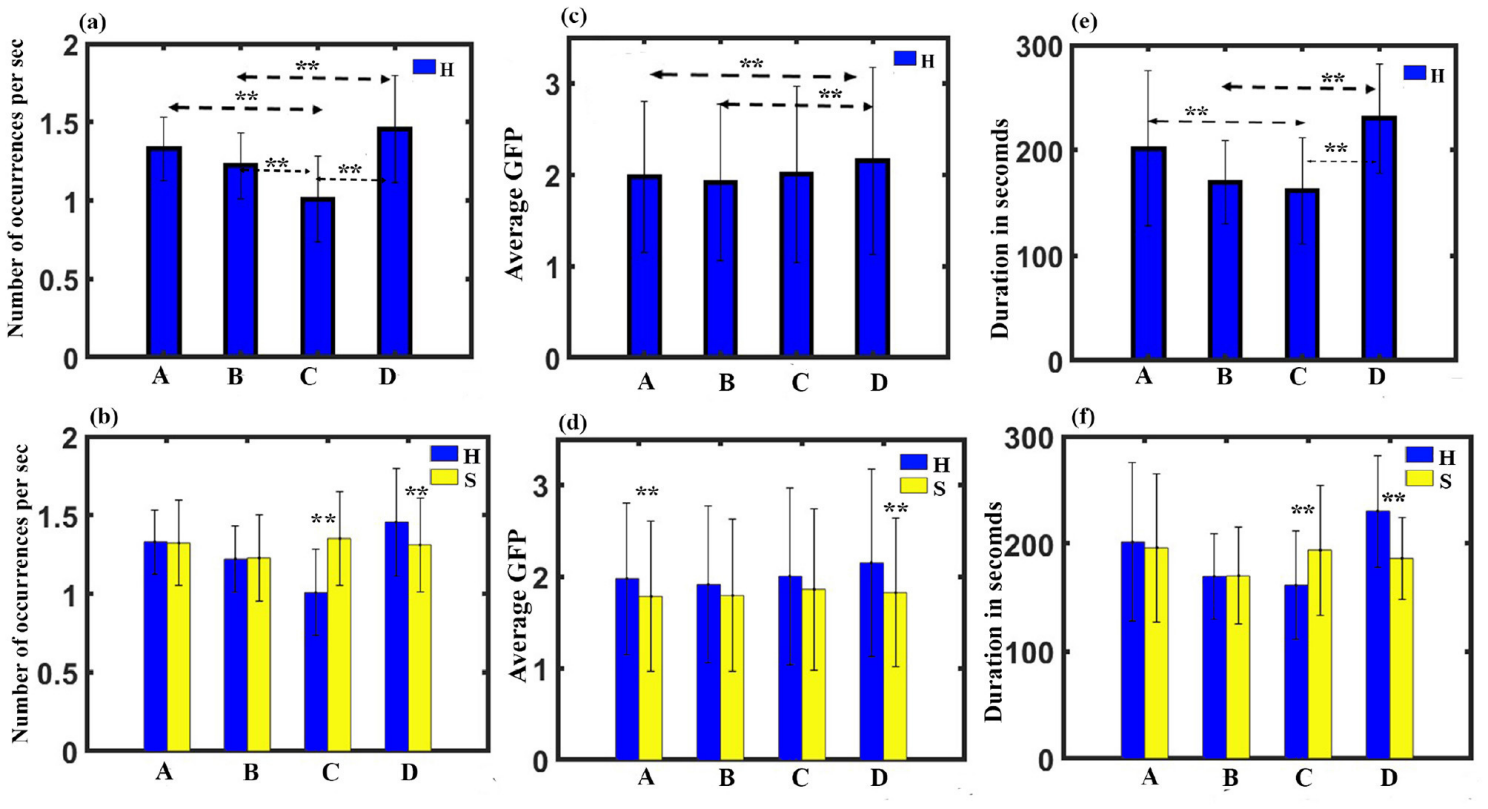
Microstate parameters. **(A)** Relative frequency of occurrence of microstates during Happy stimulus across gender. **(B)** Relative frequency of occurrence in each microstate during Happy and Sad stimulus across gender. **(C)** Relative GFP of microstates during Happy stimulus across gender. **(D)** Relative GFP in each microstate during Happy and Sad stimulus across gender. **(E)** Relative duration of microstates during Happy stimulus across gender. **(F)** Relative duration in each microstate during Happy and Sad stimulus across gender (**FDR corrected, *p* < 0.05; error bars = 1 SD).

**Figure 4:**
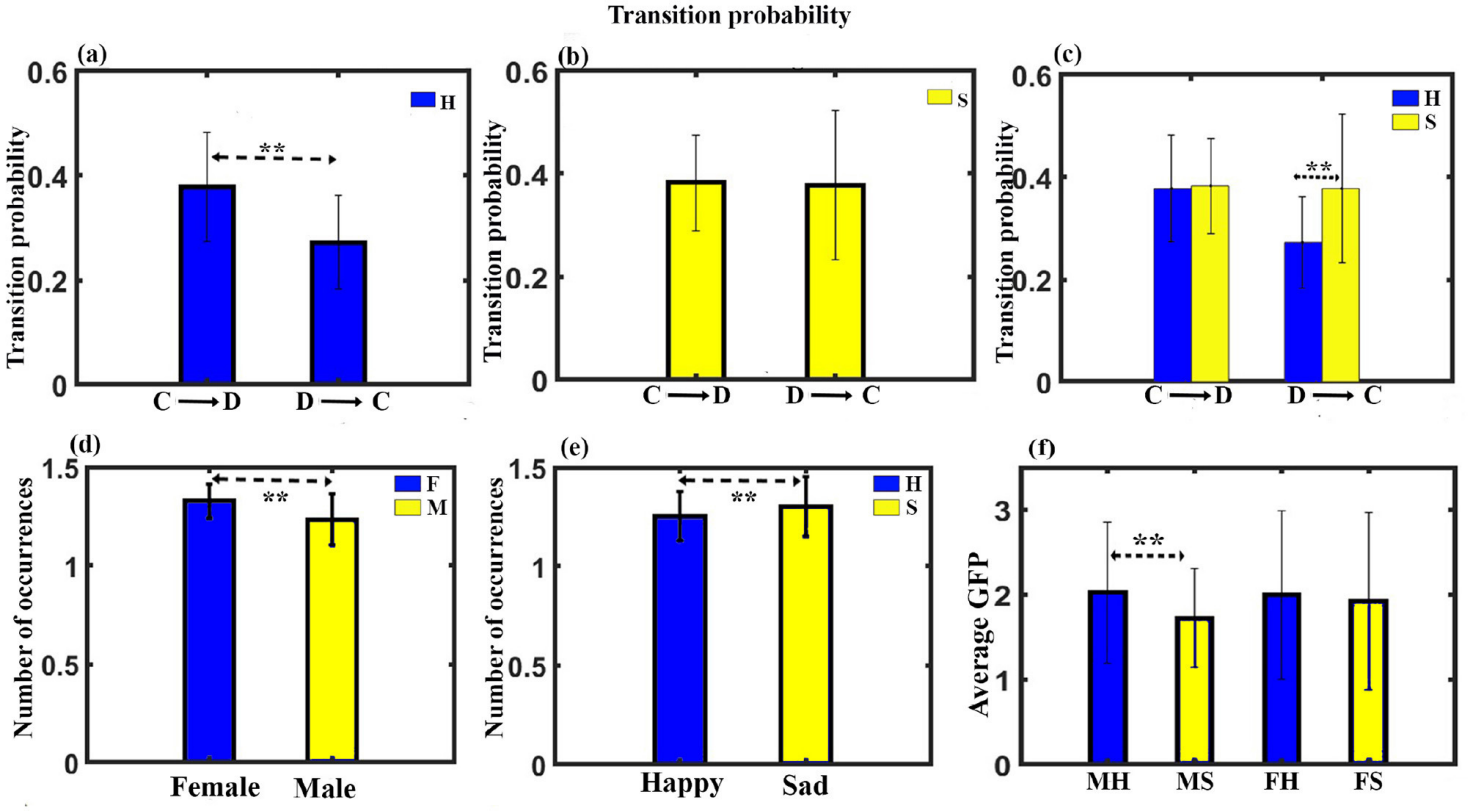
Microstate parameters. **(A)** The relative transition probability between class C and class D while listening to happy music. **(B)** The relative transition probability between class C and class D while listening to sad music. **(C)** The relative transition probability from class C to class D and from class D to class C while listening to happy and sad music listening. **(D)** Relative mean occurrence of microstates for female and male participants. **(E)** Relative mean occurrence of microstates while listening to happy and sad music listening. **(F)** Relative mean GFP of microstates while listening to happy and sad music listening for male and female participants (**FDR corrected, *p* < 0.05; error bars = 1 SD).

**Figure 5:**
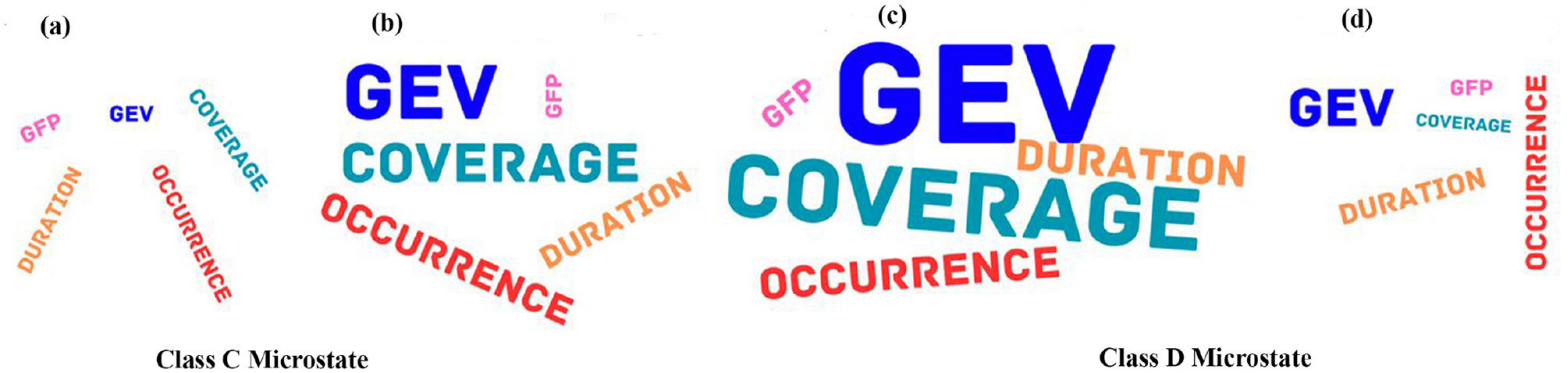
Word cloud of microstate features during happy and sad music listening. **(A)** Class C brain microstate features during happy music. **(B)** Class C brain microstate features during sad music. **(C)** Class D brain microstate features during happy music. **(D)** Class D brain microstate features during Sad music. Font size of the words is proportional to value of the Microstate features.

(3) Occurrence analysis: A three-way ANOVA with gender as in between factor, stimulus, and microstate as within factor was administered. The results show no significant three-way interaction. We obtained a significant two-way interaction between stimulus and microstate with a Greenhouse-Geisser correction (*F*_1.748, 52.451_ = 13.9, *p* < 0.001).To examine the simple effect of microstates, a one-way repeated measures ANOVA was conducted. Findings revealed a significant effect of microstates on frequency of occurrence for happy stimulus with (*F*_3, 93_ = 16.089, *p* < 0.001). Further *post-hoc* pairwise comparison with FDR correction revealed class D state to be significantly higher than class C (t = 5.2046, df = 31, *p* < 0.001, effect size = 0.9201) and class B (t = 4.1262, df = 31, *p* < 0.001, effect size = 0.7294) during the happy stimulus. We also found the class C state to be significantly reduced than class A (t = −5.0341, df = 31, *p* < 0.001, effect size = −0.8899) and class B (t = −3.2324, df = 31, *p* < 0.05, effect size = −0.5714), as shown in Figure 3A. We also obtained microstate class C and class D during the sad stimulus to be significantly higher and lower than the class C (t = 4.9191, df = 31, *p* < 0.001, effect size = 0.8696) and class D (t = −2.4689, df = 31, *p* < 0.05, effect size = −0.4365), respectively, during the happy stimulus as shown in Figure 3B.

(4) GFP analysis: A three-way ANOVA with gender as in between factor, stimulus, and microstate as within factor was administered. The results show no significant three-way interaction. We obtained a significant two-way interaction between stimulus and microstate with a Huynh-Feldt correction (*F*_2.674, 80.216_ = 0.431, *p* < 0.05). One-way repeated measure ANOVA was done to investigate the simple effect of microstates. Findings revealed a significant effect of microstates on GFP for happy stimulus with (*F*_3, 93_ = 5.163, *p* < 0.010). Further *post-hoc* pairwise comparison with FDR correction revealed class D state to be significantly higher than class B (t = 3.3277, df = 31, *p* < 0.05, effect size = 0.5883) and class A (t = 2.5716, df = 31, *p* < 0.05, effect size = 0.4546) during the happy stimulus (Figure 3C). We also obtained microstate class A and class D during the sad stimulus to be significantly lower than the class A (-t = 2.7627, df = 31, *p* < 0.05, effect size = −0.4884) and class D (t = −5.0481, df = 31, *p* < 0.001, effect size = −0.8924), respectively, during the happy stimulus as shown in Figure 3D.

(5) Duration Analysis: A three-way ANOVA with gender as in between factor, stimulus, and microstate as within factor was administered. The results show no significant three-way interaction. We obtained a significant two-way interaction between stimulus and microstate with a Greenhouse-Geisser correction (*F*_1.638, 49.135_ = 8.384, p = 0.001). To examine the simple effect of microstates, a one-way repeated measures ANOVA was conducted. The findings revealed a significant effect of microstates on duration for happy stimulus with a Greenhouse-Geisser correction (*F*_2.130, 66.040_ = 8.746, *p* < 0.001). Further *post-hoc* pairwise comparison with FDR correction revealed class D state to be significantly higher than class C (t = 4.4648, df = 31, *p* < 0.001, effect size = 0.7893) and class B (t = 5.2560, df = 31, *p* < 0.001, effect size = 0.9291) during the happy stimulus. We also found the class C state to be significantly reduced than class A (t = −2.4875, df = 31, *p* < 0.05, effect size = −0.4397), as shown in Figure 3E. We also obtained microstate class C and class D during the sad stimulus to be significantly higher and lower than the class C (t = 2.4471, df = 31, *p* < 0.05, effect size = 0.4326) and class D (t = −4.0303, df = 31, *p* < 0.005, effect size = −0.7125), respectively, during the happy stimulus as shown in Figure 3F.

(7) Transition probability: Transition probability between the class C and class D microstates for all the four conditions was further analyzed. A three-way ANOVA with gender as in between factor, stimulus, and microstate as within factor was administered. The results show no significant three-way interaction. We obtained a significant two-way interaction between stimulus and microstate for transition probability (*F*_11, 330_ = 6.637, *p* < 0.001). Further *post-hoc* pairwise comparison with FDR correction revealed a significant enhancement for class C to class D transition compared to class D to class C (t = 5.4284, df = 31, *p* < 0.0001, effect size = 0.9596) during happy stimulus (Figures 4A, B). We also found a significant class D to class C transition during the sad stimulus compared to the happy stimulus (t = 3.7808, df = 31, *p* < 0.0001, effect size = 0.6684), as shown in Figure 4C.

(8) Mean occurrence analysis: We applied mixed ANOVA to study the effect of gender and musical stimulus on the mean frequency of occurrence and did not find a significant interaction effect between gender and musical stimulus. However, the main effect of gender (*F*_1, 30_ = 5.924, p = 0.021) and the stimulus (*F*_1, 30_ = 4.155, *p* = 0.05) was statistically significant as shown in Figures 4D, E.

(9) Mean GFP: We applied mixed ANOVA to study the effect of gender and musical stimulus on the mean GFP and found a significant interaction effect between gender and musical stimulus with (*F*_1, 30_ = 4.786, *p* = 0.037). Further *post-hoc* analysis revealed male during happy stimulus had significantly enhanced mean GFP than during sad stimulus (t = 3.3941, df = 17, *p* < 0.005, effect size = 0.8232) as shown in Figure 4F. Figure 5 presents a word cloud depicting the brain microstate features of classes C and D during happy and sad music listening.

### 3.3 CSD and functional connectivity analysis

**Figure 6:**
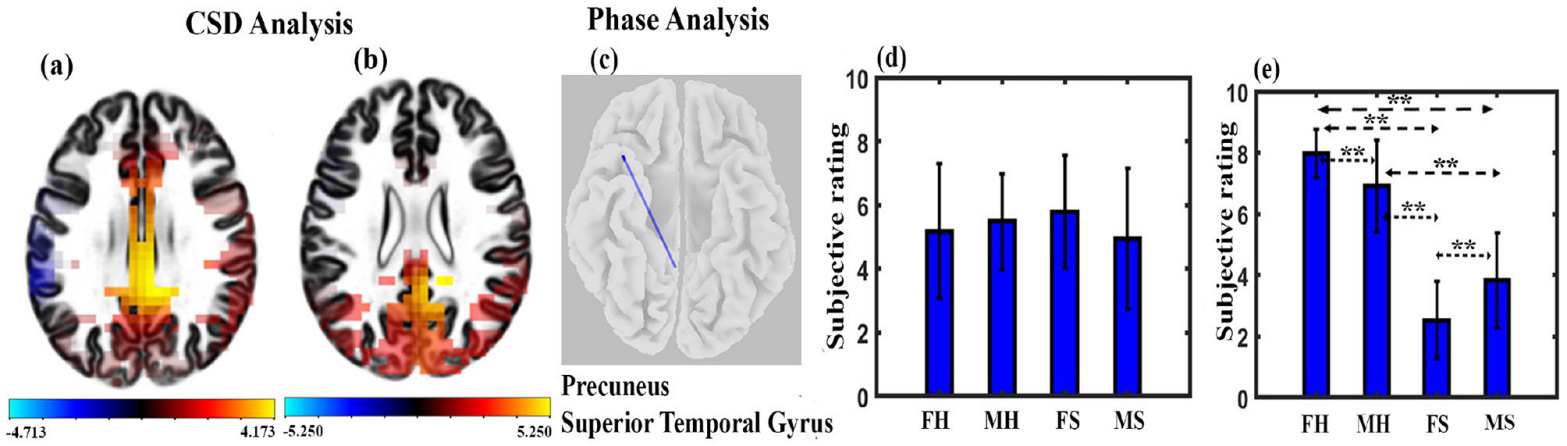
Microstate maps. **(A)** Brain regions depicting the CSD change in the alpha band under MH and MS state (*p* < 0.05). **(B)** Brain regions depicting the CSD change in the alpha band under FH and FS state at *p* < 0.05. **(C)** Brain regions depicting the phase coherence changes in the alpha band under FH and FS state (*p* < 0.05). **(D)** Mean subjective rating for arousal by the participants under different conditions. **(E)** Mean subjective rating for valence by the participants under different conditions (**FDR corrected, *p* < 0.05; error bars = 1 SD).

We calculated CSD for 6,239 voxels of the brain and found significant differences across stimulus conditions, as shown in Figure 6. Figure 6A shows several brain regions (67) to have significant higher brain activity across the MH condition as compared to the MS condition, with *t*-values ranging from 4.236 to 4.712 at *p* < 0.05 and effect size ranging from 1.0274 to 1.11428. They were cingulate gyrus, posterior cingulate, precuneus, and paracentral lobule. One hundred and fourteen regions were found to have significantly higher brain activity during the FH state compared to FS, as shown in Figure 6B. Most of the regions were located upon cingulate gyrus, precuneus, posterior cingulate, parahippocampal gyrus, and cuneus, with a *t*-value ranging from 3.817 to 5.25 at *p* < 0.05 and effect size ranging from 0.9855 to 1.3555. We performed lagged phase coherence analysis across 68 × 68 brain regions defined by the standard Desikan-Killiany atlas (Supplementary Table S1). The result shows that a connection between precuneus and superior temporal gyrus has significantly reduced connectivity (t = 5.88, df = 14, *p* < 0.05, effect size = 1.5182) during the FH state as compared to the FS state, as shown in Figure 6C.

### 3.4 Arousal and valence analysis

We performed arousal and valence analysis for the two stimuli for both male and female participants. The mean arousal ratings of the participants are shown in Figure 6D. A mixed ANOVA with gender as between factor and musical stimulus as within factor was administered. The interaction effect or main effect was not significant. We applied a mixed ANOVA gender as between factor and musical stimulus as within factor for valence and found a significant interaction effect between gender and musical stimulus (*F*_1, 30_ = 14.468, *p* < 0.001). The mean valence ratings of the participants are shown in Figure 6E. Further *post-hoc* analysis with FDR correction shows that FH was significantly higher than FS (t = 13.6954, df = 14, *p* < 0.001, effect size = 3.5361), MS (t = 9.3009, df = 30, *p* < 0.001, effect size = 3.29), and MH (t = 2.4329, df = 30, *p* < 0.05, effect size = 0.86). MH was also significantly higher than FS (t = 8.8686, df = 30, *p* < 0.001, effect size = 3.14) and MS (t = 6.6193, df = 16, *p* < 0.001, effect size = 1.6548). We also found that FS was significantly different than MS (t = 2.5890, df = 30, *p* < 0.05, effect size = 0.92).

## 4 Discussion

Happy music enhances cognitive functions such as attention and spatial skills, while sad music aids emotional processing and resilience. However, limitations in scientific standardization and real-time brain analysis have restricted our understanding of music's effects. This study seeks to address these gaps through microstate analysis. Specifically, we examined the alpha band microstates that underlie the basic emotions of happiness and sadness in male and female participants evoked through music videos. We analyzed several features of microstates, including GEV, coverage, occurrence, duration, and transition probability, for FH, FS, MH, and MS states. We also conducted EEG source-level analysis of the brain using eLoreta and compared CSD and functional connectivity during these conditions.

Earlier studies examining cognitive enhancement, such as increased alertness from listening to happy music, commonly attribute the effect to heightened arousal and mood, known as the Arousal and Mood hypothesis (Thompson et al., 2001; Husain et al., 2002). Further research reveals that happy music directly activates centers associated with attention (Fernandez et al., 2019; Putkinen et al., 2017) and intelligence (Gupta et al., 2018; Jaušovec and Habe, 2003). Similarly, sadness evoked by sad music is linked to empathy (Vuoskoski and Eerola, 2012; Huron and Vuoskoski, 2020) and autobiographical memories (Taruffi and Koelsch, 2014; Gupta et al., 2023) as the most prominent factor underpinning sadness.While cumulative effects during the music listening on cognitive and emotional processes have been explored, the dynamic aspect of these effects is often overlooked. This investigation advances the study by examining the dynamic nature of music's impact during the course of listening through microstate analysis.

In our initial analysis, we aim to identify the four microstates for FH, FS, MH, and MS conditions. We obtain four microstates optimally explaining (GEV) for the four states separately as shown in Figure 1. The results indicate that the topography of these four microstates are similar to the classical four microstates identified in previous studies (Pascual-Marqui et al., 2014; Gu et al., 2022) including an earlier microstate study involving the current DEAP dataset (Hu et al., 2023) (Supplementary Figures S3–S5) and have an overall high spatial correlation among the corresponding class A-D microstates for all the conditions.

We further proceeded to examine various parameters, including GEV, coverage, occurrence, GFP, duration, and transition probability. Findings show that happy music video is linked with a significantly higher presence (GEV), increased total amount of time (coverage), higher frequency (occurrence), greater brain activity (GFP), and longer average life span (duration) of the class D microstate compared to sad music video. Conversely, sad music video is associated with a significantly higher presence, increased total amount of time, higher frequency, and longer duration of the class C microstate compared to happy music video (Figures 2, 3). Moreover, the analysis indicates that microstate class D is notably heightened, whereas class C is downregulated during happy music videos, in contrast to other microstates. Further analysis was conducted on the transition probability between microstates class C and class D for both happy and sad stimuli. The results indicated that happy music upregulated class D microstate and downregulated class C microstate as shown in Figures 4A, B, while sad music had the opposite effect as shown in Figure 4C. The findings are consistent across both genders.

Microstates class C and class D are linked to enhanced DMN and attention, respectively, as observed in earlier studies (Khanna et al., 2015; Michel and Koenig, 2018; Koenig et al., 2002). The DMN has been associated with mind-wandering (Mason et al., 2007; Kucyi et al., 2013), and an enhanced DMN activity during sad music listening has been linked to an increased mind-wandering (Taruffi et al., 2017). Thus, in line with earlier findings (Taruffi et al., 2017; Gupta et al., 2018; Putkinen et al., 2017; Gupta et al., 2023; Husain et al., 2002), the analysis of microstate parameters reveals that regardless of gender: (1) the attentiveness is significantly larger during happy music video than during sad music video, (2) conversely, mind-wandering is significantly higher during sad music video than during happy music video, and (3) during happy music video, the brain exhibits increased attentiveness and a decreased mind wondering. Figure 5 illustrates a word cloud of brain microstate features for classes C and D during happy and sad music listening, showcasing musical stimuli-based differences as revealed by EEG analysis.

Traditional EEG analyses, such as power and phase connectivity, typically portray an apparent continuous activation of brain regions throughout the duration of music listening. Microstate analysis reveals that functional brain states, such as enhanced attention, are not continuously present during music listening; instead, they manifest as brief episodes lasting tens of milliseconds. These microstates represent fundamental instantiations of human neurological tasks, and further analysis elucidates the duration, frequency, potential, and prominence of these microstates during music listening.

Furthermore, our findings revealed that the GFP for class A microstate was significantly higher during the happy state compared to the sad state, regardless of gender (Figure 3D). This suggests that the happy state is characterized by enhanced auditory processing as class A microstate is linked with the auditory network (Khanna et al., 2015; Michel and Koenig, 2018; Koenig et al., 2002; Tarailis et al., 2023). This finding supports the notion of increased awareness of music during happy music listening, which has been reported in a previous study (Taruffi et al., 2017). Furthermore, regardless of gender, the GEV for the class B microstate was significantly higher during the sad state compared to the happy state (Figure 2B). The class B microstate is associated with visual processing, self-visualization, autobiographical memory, and scene visualization (Tarailis et al., 2023). This observation likely supports enhanced spontaneous self-referential processes and thoughts that are enriched with images during the sad music listening as reported in previous study (Taruffi et al., 2017). However, further research is necessary to gain a more comprehensive understanding of both these relationships and delve deeper into their implications.

The results also revealed that regardless of musical stimuli, female participants had significantly larger average life span for the class D microstate compared to other microstates of female participants and to the class D microstate of male participants (Figures 2E, F).

We also analyzed mean value of parameters of GEV, coverage, occurrence, GFP, and duration. The results indicated that regardless of musical stimuli, the mean frequency of occurrence of microstates was significantly higher in female participants compared to male participants (Figure 4D). The finding is in support of earlier studies (Whittle et al., 2011; Al-Fahad and Yeasin, 2019) that showed that males tend to exhibit a greater likelihood of remaining in a specific state, while females tend to exhibit a greater likelihood of being in transient states. Similarly, we observed that the mean frequency of occurrence of microstates was significantly higher during sad music listening compared to listening to happy music regardless of gender (Figure 4E). Mean GFP analysis shows brain during happy music listening has higher electrical activity than the brain during sad music listening (Figure 4F) for the male participants.

EEG microstate analysis reveals that happy and sad music evoke distinct patterns in GEV, duration, GFP, and occurrence across genders. However, gender notably impacts parameters such as coverage, mean occurrence, and mean GFP indicating that both emotional content and gender significantly influence neural responses to music.

CSD reflects the mean brain activity during the musical videos. CSD analysis revealed that the happy state was characterized by enhanced activity in the central-parietal regions compared to the sad state across gender (Figures 6A, B). The higher CSD activity observed during the happy state compared to the sad state aligns with previous research that has found a positive correlation between the valence of the music stimulus and brain activity (Koelstra et al., 2011). Additionally, studies have shown that an increased activity in the central-parietal regions during music listening is associated with enhanced attentiveness (Markovic et al., 2017; Jäncke et al., 2015; Gupta et al., 2023). Thus, findings suggest an enhanced attention during happy state compared to sad state. The current CSD analysis finding is in line with the results of the microstate analysis and likely reflect the predominant characteristics of the brain state during the whole period of happy music video compared to sad music video.

According to the Lagged Phase Synchronization analysis, the brain exhibited greater connectivity between the precuneus and superior temporal gyrus (STG) during the sad state compared to the happy state for female participants (Figure 6C). Enhanced functional connectivity between STG and precuneus represents the increased connection between the auditory cortex and DMN. The finding is consistent with a previous study (Taruffi et al., 2017) which found increased centrality in the DMN regions (including the precuneus) during sad music listening compared to happy music listening. The author suggested that increased centrality leads to enhanced DMN activity and thus increased mind wandering during sad music listening. Therefore, the enhanced brain connectivity observed between the precuneus and STG during the sad state could be related to increased DMN activity during sad music video. The result is in line with the findings of the microstate analysis. However, further research is needed to confirm this relationship.

EEG source localization studies for musical stimuli are infrequent, with the majority relying on neuroimaging techniques such as fMRI and PET. Consistent findings indicate that compared to unpleasant music, pleasant music activates specific regions, including the subcallosal cingulate, inferior frontal gyrus, anterior insula, parietal operculum, and ventral striatum. Conversely, the unpleasant scrambled condition shows heightened activity in the amygdala, hippocampus, and temporal poles (Mitterschiffthaler et al., 2007; Blood et al., 1999; Trost et al., 2012). However, eLoreta-based source analysis faces challenges in spatial resolution, particularly for deeper emotion processing regions such as the amygdala, insula, and hippocampus. While our CSD analysis aligns with previous EEG findings (Markovic et al., 2017; Jäncke et al., 2015; Koelstra et al., 2011), further research is essential to establish correlations between fMRI and EEG studies. Future investigations concurrently utilizing both modalities promise deeper insights. Moreover, we utilized a 32-channel setup for source localization analysis. Studies with a greater number of electrodes would be invaluable in gaining deeper and more precise insights into the underlying neural sources during music video listening.

Behavioral analysis shows that there was no difference in the arousal rating of the participants in any conditions (Figure 6D). Valance analysis indicates that the FH state had a significantly higher positive state followed by the MH state compared to FS and MS states (Figure 6E). FS state participants had particularly the highest sad experience compared to other states.

Music conveys emotions that are perceived by listeners, giving rise to two contrasting viewpoints: the “cognitivist” position, asserting that music expresses emotions perceived by the listener, and the “emotivist” position, suggesting that music also elicits emotions (Juslin et al., 2001). In a seminal work, Gabrielsson (2020) proposed various relationships between perception and induction, including positive, negative, no systematic relationship, and no relation. While some researchers assume a positive relationship, it is not universally applicable. It is crucial to distinguish between perceiving an emotion in music and actually experiencing an emotional response to it. Statistically equal ratings of felt and expressed emotion occur when music is liked, as opposed to disliked by participants (Schubert, 2010, 2013). The current investigation shows a high rating for liking for both music videos (Supplementary Figure S6), suggesting a smaller gap between felt and expressed emotion. However, future studies that distinguish and comparatively analyze both aspects are crucial.

Key implications of EEG microstate analysis on brain cognitive functions during music listening include: (1) Whole-Brain Integration: Microstate analysis reveals that cognition emerges from coordinated brain networks rather than isolated regions, supporting the global workspace theory (Baars et al., 2021) that cognitive processes result from integrated brain activity across distributed areas. (2) Temporal Dynamics: Microstate analysis introduces a temporal aspect, showing that cognitive functions unfold through brief, shifting brain states. This aligns with dynamic cognition theories, which suggest that mental processes depend on transient network configurations, enhancing models of sequential processing. (3) Simultaneous Function Integration: By linking distinct microstates to different cognitive functions, microstate analysis shows that multiple functions operate simultaneously, supporting the view that experiences are shaped by various interacting processes for cohesive perception and response.

**Figure 7:**
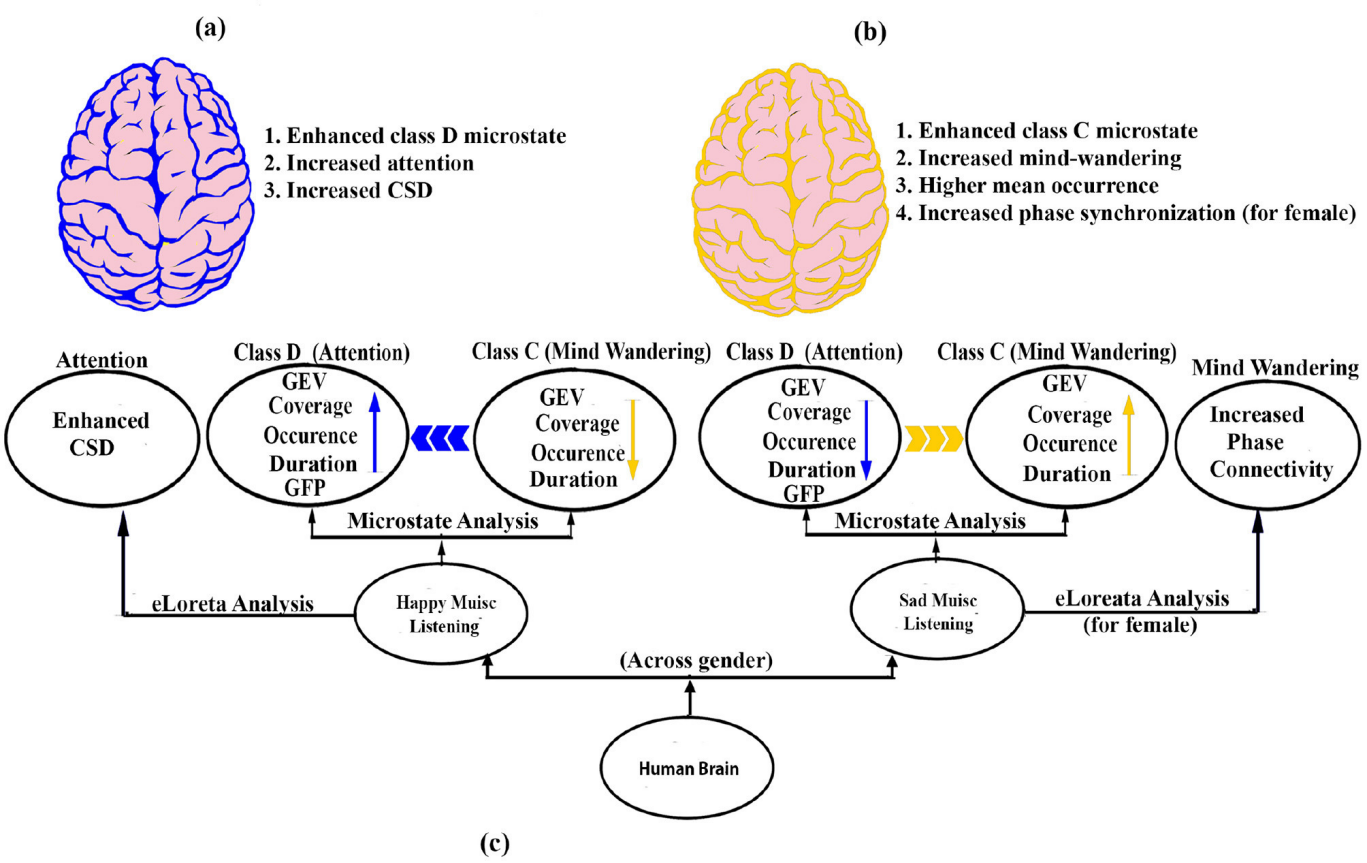
Schematic model. **(A)** Brain during happy music is marked by enhanced attention (blue color). **(B)** Brain during sad music is marked by enhanced mind wandering (yellow color). **(C)** Potential neural pathways that enhances attention and mind wandering during happy and sad music listening, respectively.

In summary (Figure 7), our results demonstrate that regardless of gender, the microstate characteristics of the brain during happy and sad music listening are unique and distinct from each other. Specifically, happy music listening is associated with enhanced class D, indicating increased attentiveness, while sad music listening is associated with higher class C, indicating increased mind-wandering. The results are in line with an earlier study investigating the effect of happy and sad music on mind wandering and meta awareness (Taruffi et al., 2017). However, gender significantly affects coverage, mean GFP, and mean occurrence, indicating that both emotion and gender shape neural responses to music. We also found a significantly enhanced mean occurrence of microstates during sad music listening compared to happy one. The results of the eLoreta analysis also support the results of the microstate analysis.

## 5 Limitations and future directions

Although our study provides insight into the relationship between music-induced emotions and brain microstates, several limitations warrant further research. First, it remains unclear how the varying levels of happiness and sadness in music impact microstates, particularly classes C and D. Real-time subjective assessments of attention and mind-wandering could provide a more nuanced understanding. Expanding this research with a wider range of happy and sad music stimuli and different durations may enhance its generalizability. The current microstate investigation was alpha band specific analysis future comparative investigations across all frequency bands could provide further insights and expand the analysis. Further studies exploring the relationship between microstate dynamics and individual psychological traits, such as depression, empathy, and cognition, would provide deeper insights and enhance our understanding. Furthermore, the mind wandering induced by sad music could differ for individuals with conditions such as depression or PTSD, and caution is advised to use sad music therapeutically, as it may be counterproductive in these populations (van den Tol, 2016). Future studies should utilize a dense montage system with 64 or more electrodes to improve microstate and source localization analysis, particularly for identifying the neural sources underlying microstates. Additionally, the unique ability of happy music to foster attention and downregulate mind wandering could be utilized in the healthcare system.

## Data Availability

Publicly available datasets were analyzed in this study. This data can be found here: http://www.eecs.qmul.ac.uk/mmv/datasets/deap/.
