# Supplementary material for: A comparative study of EEG microstate dynamics during happy and sad music videos
Source: Front Hum Neurosci. 2025 Feb 6;18:1469468. doi: 10.3389/fnhum.2024.1469468 (PMC11841423; doi:10.3389/fnhum.2024.1469468)
Supplement: Supplementary file 1 [file Data_Sheet_1.pdf]

Supplementary Information:

A Comparative Study of EEG Microstate Dynamics During Happy and Sad Music videos.

Ashish gupta[1], Chandan Kumar Srivastava[2], Braj Bhushan[3], and Laxmidhar Behera[1,4*]

[1]Department of Electrical Engineering, Indian Institute of Technology, Kanpur, India.

[2]Department of Humanities and Social Sciences, Indian Institute of Technology, Bombay, India.

[3]Department of Humanities and Social Sciences, Indian Institute of Technology, Kanpur, India.

[4]School of Computing and Electrical Engineering, Indian Institute of Technology, Mandi, India.

*Correspondence and requests for materials should be addressed to L.D. (lbehera@iitk.ac).

1. **Co-ordinates for the 68 regions of interest used in the lag Phase synchronization analysis:**

68 ROI defined according to standard Montreal Neurological Institute (MNI) template, Brodmann areas (BA) the standard Desikan-Killiany atlas.

Table S1: -

X-MNI	Y-MNI	Z-MNI	Lobe	Structure	Brodmann area	ROI
-55	-45	5	Temporal Lobe	Middle Temporal Gyrus	Brodmann area 21	1
0	20	25	Limbic Lobe	Anterior Cingulate	Brodmann area 33	2
-45	15	50	Frontal Lobe	Middle Frontal Gyrus	Brodmann area 8	3
0	-80	20	Occipital Lobe	Cuneus	Brodmann area 18	4
-20	-10	-30	Limbic Lobe	Parahippocampal Gyrus	Brodmann area 35	5
-25	-55	-15	Occipital Lobe	Fusiform Gyrus	Brodmann area 19	6
-45	-70	30	Parietal Lobe	Angular Gyrus	Brodmann area 39	7
-55	-30	-25	Temporal Lobe	Inferior Temporal Gyrus	Brodmann area 20	8
0	-50	25	Limbic Lobe	Posterior Cingulate	Brodmann area 23	9
-45	-85	0	Occipital Lobe	Middle Occipital Gyrus	Brodmann area 19	10
-35	30	-10	Frontal Lobe	Inferior Frontal Gyrus	Brodmann area 47	11
-5	-75	0	Occipital Lobe	Lingual Gyrus	Brodmann area 18	12
-5	45	-15	Frontal Lobe	Medial Frontal Gyrus	Brodmann area 11	13
-65	-30	-15	Temporal Lobe	Middle Temporal Gyrus	Brodmann area 21	14
-20	-30	-20	Limbic Lobe	Parahippocampal Gyrus	Brodmann area 35	15
-5	-30	60	Frontal Lobe	Medial Frontal Gyrus	Brodmann area 6	16
-55	20	20	Frontal Lobe	Inferior Frontal Gyrus	Brodmann area 45	17
-45	45	-15	Frontal Lobe	Middle Frontal Gyrus	Brodmann area 11	18
-50	35	0	Frontal Lobe	Inferior Frontal Gyrus	Brodmann area 47	19

-10	-90	5	Occipital Lobe	Cuneus	Brodmann area 17	20
-50	-25	60	Parietal Lobe	Postcentral Gyrus	Brodmann area 1	21
0	-20	40	Limbic Lobe	Cingulate Gyrus	Brodmann area 24	22
-45	-10	60	Frontal Lobe	Precentral Gyrus	Brodmann area 6	23
-10	-60	45	Parietal Lobe	Precuneus	Brodmann area 7	24
-5	40	5	Limbic Lobe	Anterior Cingulate	Brodmann area 32	25
-35	50	15	Frontal Lobe	Middle Frontal Gyrus	Brodmann area 10	26
-20	40	50	Frontal Lobe	Superior Frontal Gyrus	Brodmann area 8	27
-25	-60	65	Parietal Lobe	Superior Parietal Lobule	Brodmann area 7	28
-60	-5	0	Temporal Lobe	Superior Temporal Gyrus	Brodmann area 22	29
-60	-40	40	Parietal Lobe	Inferior Parietal Lobule	Brodmann area 40	30
-10	65	-10	Frontal Lobe	Superior Frontal Gyrus	Brodmann area 11	31
-25	15	-35	Temporal Lobe	Superior Temporal Gyrus	Brodmann area 38	32
-50	-25	10	Temporal Lobe	Transverse Temporal Gyrus	Brodmann area 41	33
-40	15	-5	Frontal Lobe	Inferior Frontal Gyrus	Brodmann area 47	34
55	-40	5	Temporal Lobe	Superior Temporal Gyrus	Brodmann area 22	35
5	20	25	Limbic Lobe	Anterior Cingulate	Brodmann area 24	36
45	15	40	Frontal Lobe	Middle Frontal Gyrus	Brodmann area 9	37
5	-80	20	Occipital Lobe	Cuneus	Brodmann area 18	38
20	-5	-30	Limbic Lobe	Uncus	Brodmann area 28	39
35	-50	-20	Temporal Lobe	Fusiform Gyrus	Brodmann area 37	40
50	-65	30	Parietal Lobe	Angular Gyrus	Brodmann area 39	41
60	-30	-25	Temporal Lobe	Inferior Temporal Gyrus	Brodmann area 20	42
0	-45	25	Limbic Lobe	Cingulate Gyrus	Brodmann area 31	43
40	-90	0	Occipital Lobe	Middle Occipital Gyrus	Brodmann area 18	44

20	40	-20	Frontal Lobe	Inferior Frontal Gyrus	Brodmann area 11	45
5	-70	0	Occipital Lobe	Lingual Gyrus	Brodmann area 18	46
5	45	-15	Frontal Lobe	Medial Frontal Gyrus	Brodmann area 11	47
65	-25	-15	Temporal Lobe	Middle Temporal Gyrus	Brodmann area 21	48
20	-30	-15	Limbic Lobe	Parahippocampal Gyrus	Brodmann area 35	49
5	-25	60	Frontal Lobe	Medial Frontal Gyrus	Brodmann area 6	50
55	15	15	Frontal Lobe	Inferior Frontal Gyrus	Brodmann area 44	51
50	40	-10	Frontal Lobe	Inferior Frontal Gyrus	Brodmann area 47	52
55	30	5	Frontal Lobe	Inferior Frontal Gyrus	Brodmann area 45	53
15	-85	5	Occipital Lobe	Cuneus	Brodmann area 17	54
50	-25	55	Parietal Lobe	Postcentral Gyrus	Brodmann area 1	55
0	-15	35	Limbic Lobe	Cingulate Gyrus	Brodmann area 23	56
45	-10	55	Frontal Lobe	Precentral Gyrus	Brodmann area 6	57
5	-55	40	Parietal Lobe	Precuneus	Brodmann area 7	58
5	40	5	Limbic Lobe	Anterior Cingulate	Brodmann area 32	59
45	45	20	Frontal Lobe	Middle Frontal Gyrus	Brodmann area 46	60
15	35	55	Frontal Lobe	Superior Frontal Gyrus	Brodmann area 8	61
15	-65	60	Parietal Lobe	Superior Parietal Lobule	Brodmann area 7	62
65	-10	0	Temporal Lobe	Superior Temporal Gyrus	Brodmann area 21	63
65	-35	35	Parietal Lobe	Inferior Parietal Lobule	Brodmann area 40	64
10	65	-15	Frontal Lobe	Medial Frontal Gyrus	Brodmann area 11	65
35	20	-40	Temporal Lobe	Superior Temporal Gyrus	Brodmann area 38	66
45	-25	10	Temporal Lobe	Transverse Temporal Gyrus	Brodmann area 41	67
45	10	-5	Sub-lobar	Insula	Brodmann area 13	68

2. **Musical stumili.**

Among the 40 music stimuli for which EEG recording was done in the Deap Dataset[i], we checked for the music videos that significantly expressed happiness through two steps : Step 1: We filtered music videos that were rated for the prominent emontion of happiness by more that 3/4th of the participants. Step 2: We applied one sample t-test to the selected videos to find if it significantly expressed happiness. we found one music video (Id number 11) was significantly rated for happiness ($t= 2.4347$, $df= 12$, $p < 0.05$) (fig 1). The rating was done based upon the Genova scale rating.

Fig S1

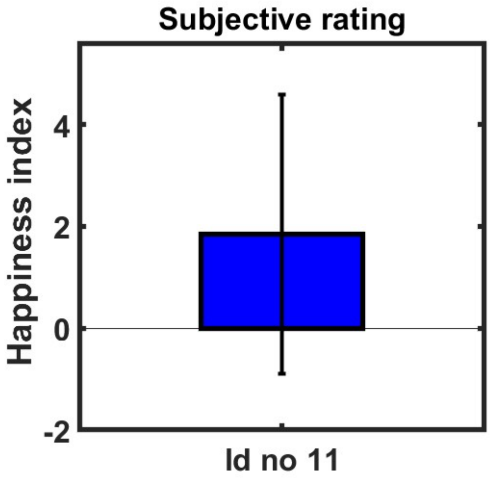

Subjective rating assesment : 1(a) Shows the subjective rating of participants mesauring the strenth of happiness elucidated by music video (id no 11) (0 = neutral, 4 = strong happiness, - 4 = some other strong emotion).

The same procedure was followed to indentitify sad music video. One music video (Id number 30) was significantly rated for sadness ($t= 2.5843$, $df= 13$, $p < 0.05$)(fig 2). The rating was done based upon the Genova scale rating.

Fig S2

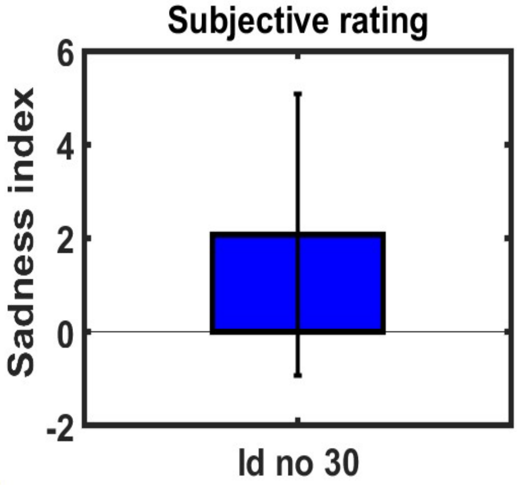

Subjective rating assesment : Shows the aboslute value for the subjective rating of participants mesauring the strenth of sadness elucidated by music video (id no 30)) (0 = neutral, 4 = strong sadness, - 4 = some other strong emotion).

These two music videos were selected for further analysis. Other music videos did not expressed significantly happiness and sadness.

Genova rating scale :

16 different emotions are arranged in a circular fashion on the Genova scale[ii]. Please note that the words provided often represent a large "emotion family" and may thus refer to a whole range of similar emotions. Thus, the Anger family also covers emotions such as rage, vexation. Joy and happy belong to same emotion family.

3. The topological orientation of the Microstate maps:

Koenig et. al.[iii] classified four microstate maps of the brain into classes A, B, C, and D according to their topological orientation (Fig S3). Specifically,
microstate map A shows a left-right orientation,
microstate map B displays a right-left orientation,
microstate map C demonstrates an anterior-posterior orientation, and
microstate map D reveals a fronto-central maximum.
Following studies have consistently adhered to this labelling convention[iv].
We categorized the acquired microstates in our study as classes A, B, C, and D based on their topographical orientation, as outlined by Koenig et. al.[iii,v] in line with earlier studies[vi,vii,viii](Fig S3-S5).

Fig S3: Four microstate maps as per the study by Koeing et. al.[iii].

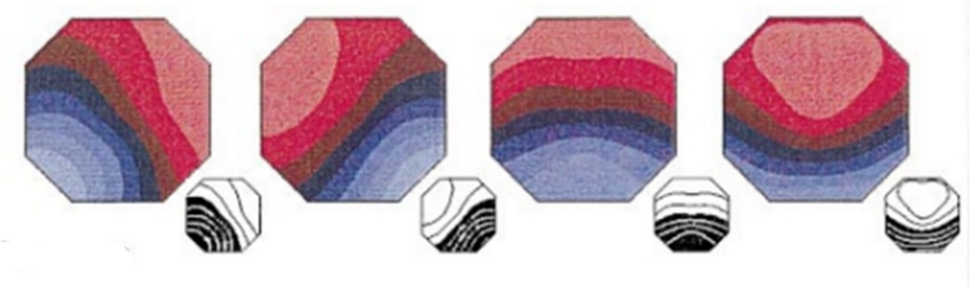

C lass A   Class B   Class C   Class D

Fig S4: Four microstate maps as per a recent study on the DEAP datasets[vi].

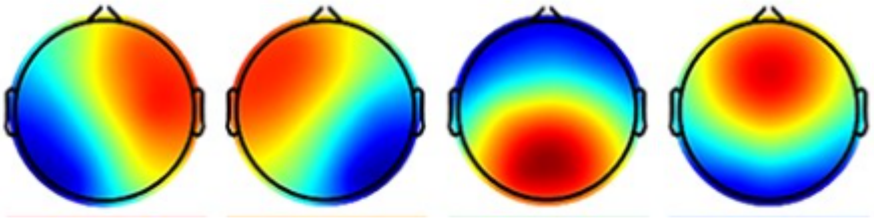

C lass A   Class B   Class C   Class D

Fig S5: Visual representation of the topography of the four canonical microstate maps, identified in numerous independent studies with varying numbers of electrodes, participants, and filter settings[iv]. Despite their distinctiveness, these four microstate maps exhibit a high level of reproducibility across different studies.

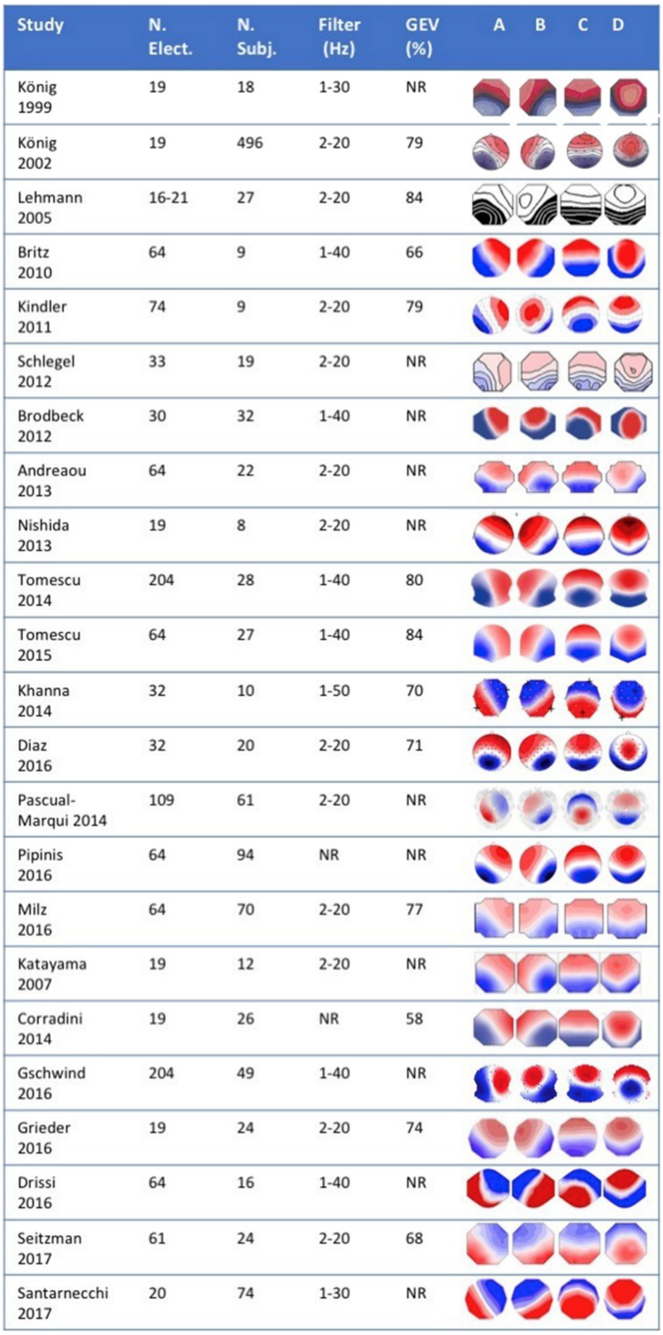

Study	N. Elect.	N. Subj.	Filter (Hz)	GEV (%)	A	B	C	D
König 1999	19	18	1-30	NR				
König 2002	19	496	2-20	79				
Lehmann 2005	16-21	27	2-20	84				
Britz 2010	64	9	1-40	66				
Kindler 2011	74	9	2-20	79				
Schlegel 2012	33	19	2-20	NR				
Brodbeck 2012	30	32	1-40	NR				
Andreaou 2013	64	22	2-20	NR				
Nishida 2013	19	8	2-20	NR				
Tomescu 2014	204	28	1-40	80				
Tomescu 2015	64	27	1-40	84				
Khanna 2014	32	10	1-50	70				
Diaz 2016	32	20	2-20	71				
Pascual-Marqui 2014	109	61	2-20	NR				
Pipinis 2016	64	94	NR	NR				
Milz 2016	64	70	2-20	77				
Katayama 2007	19	12	2-20	NR				
Corradini 2014	19	26	NR	58				
Gschwind 2016	204	49	1-40	NR				
Grieder 2016	19	24	2-20	74				
Drissi 2016	64	16	1-40	NR				
Seitzman 2017	61	24	2-20	68				
Santarnecchi 2017	20	74	1-30	NR				

Four microstates' maps obtained in the current study on the DEAP dataset is in line with four microstate maps obtained in an earlier study on the same DEAP dataset[vi] and other earlier studies[iii,iv,vii,viii]. Thus, validating the microstates obtained in the current study.

4. Subjective rating of Music videos on Linking scale

Fig S6: Subjects rated the music vedio on the scale of 1 to 9 (How much do you like the video). We found mean rating of the music video by the participants were above 6 (on a scale of 1 to 9) signifying that the music videos were positively liked by the participants.

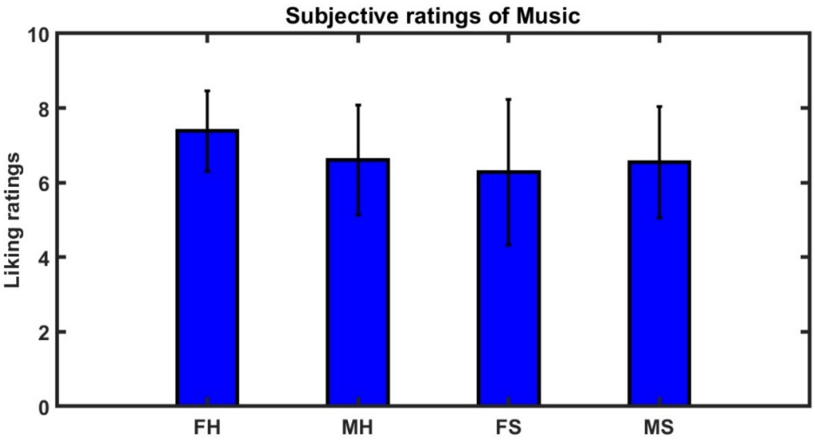

Liking rating under four conditions (1) Female during listening to happy music (FH), (2) Female during listening to sad music (FS), (3) Male during listening to happy music (MH), and (4) Male during the listening of sad music (MS).

References:

[i] Koelstra, S. et al. Deap: A database for emotion analysis; using physiological signals. IEEE transactions on affective computing 3, 18–31 (2011)

[ii] Sacharin, V., Schlegel, K., & Scherer, K. R. (2012). Geneva emotion wheel rating study. *Center for Person, Kommunikation, Aalborg University, NCCR Affective Sciences. Aalborg University, Aalborg*.

[iii] Koenig, T., Lehmann, D., Merlo, M. C., Kochi, K., Hell, D., & Koukkou, M. (1999). A deviant EEG brain microstate in acute, neuroleptic-naive schizophrenics at rest. *European archives of psychiatry and clinical neuroscience*, *249*, 205-211.

[iv] Michel, C. M., & Koenig, T. (2018). EEG microstates as a tool for studying the temporal dynamics of whole-brain neuronal networks: a review. *Neuroimage*, *180*, 577-593.

[v] Koenig, T., Prichep, L., Lehmann, D., Sosa, P. V., Braeker, E., Kleinlogel, H., ... & John, E. R. (2002). Millisecond by millisecond, year by year: normative EEG microstates and developmental stages. *Neuroimage*, *16*(1), 41-48.

[vi] Hu, W., Zhang, Z., Zhao, H., Zhang, L., Li, L., Huang, G., & Liang, Z. (2023). EEG microstate correlates of emotion dynamics and stimulation content during video watching. *Cerebral Cortex*, *33*(3), 523-542.

[vii] Liu, H., Tang, H., Wei, W., Wang, G., Du, Y., & Ruan, J. (2021). Altered peri-seizure EEG microstate dynamics in patients with absence epilepsy. *Seizure*, *88*, 15-21.

[viii] Pal, A., Behari, M., Goyal, V., & Sharma, R. (2021). Study of EEG microstates in Parkinson's disease: a potential biomarker?. *Cognitive Neurodynamics*, *15*, 463-471.
